# Author’s Response to Letter on “Willingness to Pay for HIV Prevention Commodities Among Key Population Groups in Nigeria”

**DOI:** 10.9745/GHSP-D-23-00005

**Published:** 2023-02-28

**Authors:** Olawale Durosinmi-Etti, Emmanuel Kelechi Nwala, Funke Oki, Akudo Ikpeazu, Emmanuel Godwin, Paul Umoh, Arome Shaibu, Alex Ogundipe, Abiye Kalaiwo

**Affiliations:** a JSI Research and Training Institute Inc, Abuja, Nigeria.; b National Agency for the Control of AIDS, Abuja, Nigeria.; c National AIDS and STIs Control Program, Federal Ministry of Health, Abuja, Nigeria.; d Heartland Alliance Nigeria, Abuja, Nigeria.; e U.S. Agency for International Development Nigeria, Abuja, Nigeria.

See related articles by Adepoju et al. and Durosinmi-Etti et al.

We would like to thank Adepoju et al.^1^ for recognizing that our study^2^ has advanced the understanding of self-reported willingness to pay (WTP) for HIV prevention commodities that could provide useful insights to help inform Nigeria’s HIV prevention policies and program planning for key populations. One of the key objectives of our study was to understand a reasonable price point that would ensure that the majority of the potential users could pay for HIV self-testing (HIVST) commodities and encourage market-shaping interventions that would help to improve access to the commodity. However, we disagree with the authors on several points.

We recognized that the contingent valuation approach is prone to exaggerated responses.^3^ We did not offer HIVST kits to the participants who were prospective buyers, and we recognized that a difference could exist between stated and observed WTP for HIV prevention commodities. However, an observed demand for HIVST would be inappropriate in the setting and for the target population. At the time of our study, awareness about this commodity was yet poor,^4^ and the commodity was distributed free of charge to the populations of this study (i.e., female sex workers and men who have sex with men) through donor funding. Therefore, we aimed to understand if they would be willing to pay when the commodity was provided at a commercial price to ensure the sustainability of programming amid dwindling donor funding. Only when the HIVST kits (or hospital-based HIV screening) are no longer available free to respondents does an observed demand for HIVST become reliable. It is unlikely that any individual would demand and pay for commodities that are provided for free, especially if there are no issues with access or quality. Therefore, a self-reported WTP was appropriate for our study respondents. An observed demand for HIVST would be more reliable and appropriate if the study involved the general population who currently pay for HIVST at retail outlets, as they do not benefit from free HIVST kits in Nigeria.

We used the guidance provided by Hanemann et al. in limiting exaggerated responses,^5^ using a double-bounded closed-ended dichotomous choice method in the estimation of WTP with follow-up questions (bids) to affirm the previous response on WTP. The benefits of this approach were corroborated by others.^6^ A thorough pretest of the questionnaire helped improve the validity and reliability of the survey instrument.

Readers should understand that, at the time of the study, the available brand of HIVST kit was sold at an average price of N1600 (US$4.20) to the public, so our study extrapolated the percentage of the participants that were willing to pay that price. We presented different hypothetical price points from N1000 (US$2.60) to N3000 (US$7.90). We reported that 32% were willing to pay N1600 (US$4.20), and 42% were willing to pay between N500–N1500 (US$1.30–US$4.00). The various price points should be considered, especially the price that a large proportion was willing to pay (N500–N1500). However, we agree that further study may be required to determine how the frequency of purchasing will affect WTP for HIVST and the maximum amount respondents are willing to pay to help arrive at a reasonable retail price point for most potential users of HIVST.

Adepoju et al. argued that our study failed to adjust for the effect of the global economic crisis and inflation instability in local currency, as well as other challenges that have mostly affected low- and middle-income countries. Our study anticipated these challenges and took care of them. For example, for the HIVST, we used a bidding method with various price points from N1000 (less than the prevailing price) to N3000 (almost double the prevailing price) per kit. Bidding with price points higher than N1600 took care of the anticipated increase in the retail price of the commodity due to inflation and fluctuation in currency exchange. HIVST is relatively new in the Nigerian market, and there is no adequate information to determine the trend in the commodity price. Therefore, any anticipated price increase can only be accommodated by using bid prices that are far higher than the market price when the study was conducted.

At the time of the study and until recently, the retail price of HIVST in Nigeria was not competitive (relatively higher than now) due to fewer brands and lack of competition. Despite inflation, the retail price has continued to decrease due to competition and the availability of a variety of brands. We anticipate that this will continue as the manufacturers and distributors rely more on economies of scale rather than units of sales. As part of the efforts to drive behavior change and increase the demand for HIVST, we collaborated with key stakeholders and the Government of Nigeria to co-create and launch a holistic National Communications Strategy to drive demand for HIVST in Nigeria.^7^

Based on all considered factors that can influence the respondents’ WTP, our study showed that the percentage of respondents who were willing to pay for HIVST reduced with an increase in the commodity price. A WTP study usually indicates the maximum amount respondents are willing to pay. Therefore, one would expect that any price increase due to the global economic crisis would lead to a decrease in WTP, except if there are efforts to improve the users’ purchasing power of the commodity or provide a subsidy to cushion the effects of the price increase. This is one of the reasons that we considered the factors that influence WTP to ensure that these factors are modified in a bid to improve WTP. Also, we considered other factors that may influence WTP and the maximum amount they are willing to pay, including age, marital status, educational level, employment status, and monthly income. Our multivariable analysis indicated an association between maximum WTP amount and employment status and monthly income.

In their argument around the lack of adjustment for inflation by our study, Adepoju et al. compared the WTP price for 32% of our respondents with the median WTP price reported by Tun et al.,^8^ which was inappropriate. Instead, they should have compared the percentage of respondents who were willing to pay US$5.50 and US$4.20 for HIVST from our study with the corresponding percentages reported by Tun et al.

While we agree that considering the effect of inflation on commodity pricing is important, we are aware that the market for HIVST in Nigeria is becoming competitive, which is expected to drive down the unit cost as the product manufacturer/distributors tend to focus more on economies of scale.

Among other limitations, our study did not collect information on the socioeconomic status of the key populations and its impact on overall purchasing power. Therefore, the current argument should rather focus on how to fill the limitations identified in our research.

As part of efforts to further drive down the price of HIVST in Nigeria, we are supporting the Government of Nigeria to work with distributors to negotiate lower prices and counter the effects of global inflation, which continues to truncate the final costs. However, based on our initial recommendations, efforts to scale up access to this essential commodity should target a subsidy (social marketing) for the 42% of the participants that were willing to pay between N500–N1500 (UD$1.30–US$4.00) while providing the commodity at a full commercial cost to the 32% that are willing to pay even with future increases.

## Notes

**First published online:** January 24, 2023.
